# 免疫检查点抑制剂治疗少见突变非小细胞肺癌疗效的研究进展

**DOI:** 10.3779/j.issn.1009-3419.2020.102.41

**Published:** 2021-01-20

**Authors:** 腾 李, 峻岭 李

**Affiliations:** 100021 北京，国家癌症中心/国家肿瘤临床医学研究中心/中国医学科学院北京协和医学院肿瘤医院 National Cancer Center/National Clinical Research Center for Cancer/Cancer Hospital, Chinese Academy of Medical Sciences and Peking Union Medical College, Beijing 100021, China

**Keywords:** 肺肿瘤, 少见突变, 免疫检查点抑制剂, Lung neoplasms, Rare mutation, Immune check point inhibitors

## Abstract

驱动基因的发现及针对驱动基因的靶向治疗已显著提高了肺癌患者的生存质量和时间，但目前对于*BRAF*、*HER2*、*MET*、*RET*等少见驱动基因改变肺癌患者的靶向药物的选择仍然较少。近年来免疫检查点抑制剂在肺癌治疗中取得了一定的疗效，但因为少见驱动基因突变的肺癌患者本身样本量少，开展大规模临床随机对照试验尚存在一定的困难，目前此类患者接受免疫检查点抑制剂治疗的疗效情况仍不明确。本文将对目前已掌握的免疫检查点抑制剂治疗*BRAF*、*HER2*、*MET*、*RET*等少见驱动基因改变肺癌患者的临床研究结果进行综述，以期在一定程度上为临床工作提供一些依据和参考。

## Abstract (en)

Over the past several decades, advances in driven targeted therapy has revolutionized the management of oncogene-driven non-small cell lung cancer (NSCLC). However, there are only a few targeted drugs available for patients with rare mutations, such as *BRAF*, *HER2*, *MET*, *RET*, *etc*. In recent years, immune checkpoint inhibitors (ICIs) have demonstrated promising benefit in NSCLC. Till now, efficacy of ICIs for NSCLC with rare mutation is largely unknown. It is fairly difficult to conduct a large formal prospective controlled trials because of the rarity of these mutation. In this article, currently available real world studies based on convincing clinical evidence will be reviewed, which will ultimately facilitate our rational use of ICIs for NSCLC with rare mutation.

非小细胞肺癌（non-small cell lung cancer, NSCLC）是我国最高发的恶性肿瘤之一^[1]^。近20年来驱动基因的发现及针对驱动基因的靶向治疗已显著提高了NSCLC患者的生存率，针对表皮生长因子受体（epidermal growth factor receptor, *EGFR*）、间变性淋巴瘤激酶（anaplastic lymphoma kinase, *ALK*）等常见的驱动基因改变已经研发出多代的靶向药物，使得这部分患者的生存时间从1年延长到3年-4年^[2]^。但是对于每种基因改变人数仅占肺癌整体1%-2%的少见驱动基因改变如*BRAF*、*MET*、*HER2*、*RET*等^[3]^，目前靶向治疗药物选择十分有限，部分获得突破性疗法认定的药物仍在临床试验阶段^[4]^，酪氨酸激酶抑制剂（tyrosine kinase inhibitor, TKI）治疗后的耐药机制尚不清楚，耐药后的治疗选择较少，迫切需要新疗法的探索和发展。

随着对肿瘤免疫逃避机制的认识，免疫治疗已成为肿瘤治疗中的重要组成部分，对于晚期驱动基因阴性的NSCLC，以程序性细胞死亡受体1（programmed cell death receptor 1, PD-1）/程序性细胞死亡受体配体1（PD ligand 1, PD-L1）单抗为主的免疫检查点抑制剂（immune checkpoint inhibitors, ICIs）治疗已经成为标准的治疗手段之一^[5-7]^。对于驱动基因阳性患者，近年来ICIs的应用也取得了一定突破，例如临床试验已经提示ICIs联合化疗或ICIs联合抗血管治疗在EGFR-TKI治疗失败的NSCLC中显示出较好的疗效^[8]^。但对于少见驱动基因突变的患者来说，因为突变本身发生率低，患者样本量少，开展大规模临床随机对照试验存在一定困难，目前此类患者接受免疫治疗的疗效情况尚不明确。本文将对目前已有的ICIs治疗*BRAF*、*MET*、*HER2*、*RET*等突变频率在1%-2%的少见驱动基因改变NSCLC患者的临床研究结果进行综述，以期在一定程度上为临床工作提供依据。

## 1 少见驱动基因组改变NSCLC的免疫相关标志物表达水平

肿瘤突变负荷（tumor mutation burden, TMB）、微卫星不稳定性（microsatellite instability, MSI）及PD-L1表达是目前常用的免疫治疗生物标志物。2019年欧洲肿瘤医学协会（European Society for Medical Oncology, ESMO）工作组通过系统评价分析提示对于NSCLC，由于MSI-high的患者比例极低，TMB、MSI及PD-L1三者均阳性的患者仅占0.5%，如果放宽标准仅要求PD-L1阳性伴TMB-high和（或）MSI-high，则这一比例可提高到12.7%，这部分患者可能是最有希望从免疫治疗中获益的肺癌患者^[9]^。

目前已有研究^[10]^提示与肺腺癌整体类似，少见驱动基因组改变的NSCLC患者仍是以微卫星稳定型（microsatellite stable, MS-S）为主。2018年一项回顾性研究（retrospective study, RTD）分析了ICIs治疗少见驱动基因组改变肺癌患者的MSI情况，研究纳入了*BRAF*、*MET*、*RET*、*HER2*、*NTRK*等多种驱动基因改变患者，47例患者全部为MS-S。对于PD-L1的表达，多项研究之间存在争议，一项*meta*分析提不同ALK、BRAF、HER2、PIK3CA状态和MET表达水平的患者之间PD-L1的表达无明显差异，但该研究少见突变纳入的研究仍较少，结果解读需要谨慎^[11]^。与MSI及PD-L1不同的是部分少见突变患者TMB突变型与野生型之间存在差异。2019年*JAMA*发表的一项纳入了4, 000余例NSCLC患者测序数据的研究^[12]^提示对于*BRAF*、*HER2*、*MET*突变型与野生型患者TMB没有统计学差异，而*RET*（4.6 mut/Mb *vs* 7.0 mut/Mb, *P*=0.004）及*ROS1*（4.0 mut/Mb *vs* 7.0 mut/Mb, *P*=0.03）突变型较野生型TMB更低，这一结果提示*RET*及*ROS1*基因组改变患者较野生型患者更难从免疫治疗中获益。

## 2 ICI用于少见驱动基因组改变NSCLC的疗效

### 2.1 ICIs治疗*BRAF*突变NSCLC的疗效

*BRAF*基因与免疫治疗的关系一直是研究者关注的重点。在第一部分中提到*BRAF*基因突变型与野生型患者TMB水平及PD-L1表达情况^[11, 12]^均没有显著差异，这一结果也体现在了ICIs疗效上。IMMUNOTARGET研究^[13]^是一项全球多中心ICIs单药治疗突变型NSCLC的真实世界研究，研究纳入551例各类突变肺癌患者，其中85%的患者接受Nivolumab治疗，中位治疗线数为二线。该研究共收集*BRAF*突变患者43例，客观有效率（objective response rate, ORR）为24.3%，疾病控制率（disease control rate, DCR）为54%，生存分析提示中位无进展生存期（progression-free survival, PFS）为3.1个月，中位总生存期（overall survival, OS）为13.6个月，其中亚组分析提示吸烟的*BRAF*突变患者PFS获益更大（中位PFS：无吸烟史*vs*有吸烟史为1.9个月*vs* 4.1个月，*P*=0.03）。同样在Rihawi等^[14]^回顾二线Nivoluma治疗*BRAF*突变患者的数据，11例患者中有1例*BRAF* V600E突变吸烟患者达到部分缓解（partial response, PR），虽然ORR较上述研究均比较低，但该例患者有超过29个月的持续缓解期。

*BRAF* V600E突变是最常见的*BRAF*突变类型，约占*BRAF*突变人群的50%^[15]^。在IMMUNOTARGET研究中未进一步报告不同突变类型ORR的具体结果，对于这一问题在其他研究中有所提示。在RTD研究中5例V600E突变患者（仅4例具有疗效评价）的ORR为25%，而5例非V600E突变患者的ORR为20%^[10]^，但该研究中两个亚组人数较少，在另外两项研究中扩大样本后获得相反结果。在GFPC研究中26例V600E突变患者ICIs治疗ORR为26.1%，DCR为60.9%，18例非V600E突变患者ORR为35.3%，DCR为52.9%^[16]^。同样另一项研究^[17]^回顾性分析了39例*BRAF*突变患者ICIs治疗疗效，12例有疗效评价的V600E突变患者ORR为25%，9例非V600E突变患者ORR为33%。结合上述结果来看，还是倾向于*BRAF* V600E突变患者较非V600E突变患者ICIs治疗ORR低，但这三项研究均未进一步检验两组之间差异是否有统计学意义。

那么对于这种不同突变类型的ORR能否转化为生存获益的差异呢？遗憾的是已有两项回顾性研究结果均提示*BRAF* V600E突变与非V600E突变患者的中位PFS与OS均无统计学差异。在IMMUNOTARGET研究^[13]^中，V600E突变患者的中位PFS为1.8个月，中位OS为8.2个月，非V600E突变患者的中位PFS为4.1个月，中位OS为17.2个月，两组患者中位PFS（*P*=0.20）及中位OS（*P*=0.28）无明显差异。同样另一项研究^[17]^也提示不同突变亚型组间中位PFS（*P*=0.37）和中位OS（*P*=0.53）无明显差异。

### 2.2 ICIs治疗*MET*基因改变NSCLC的疗效

*MET*基因定位于人类第7号染色体，表达为单次跨膜的受体酪氨酸激酶。其配体HGF通过与MET结合可激活下游信号通路^[18]^。2018年Sabari等^[19]^在*Annals of Oncology*发表了一项基于147例*MET* *14*突变NSCLC的回顾性研究，结果提示37%的患者PD-L1表达为0%。与*JAMA*发表研究结果不同，该研究中*MET* *14*突变型患者TMB显著低于野生型患者（3.8 mut/Mb *vs* 5.7 mut/Mb, *P* < 0.001）。在该研究中24例*MET 14*突变患者接受ICIs治疗（22例采用PD-1/PD-L1单抗，2例采用PD-1单抗联合CTLA-4单抗）ORR为17%，中位PFS为1.9个月，中位OS为18.2个月^[19]^。

与以上结果类似，在IMMUNOTARGET研究^[13]^中，36例*MET*基因组改变（13例*MET*扩增，23例*MET 14*突变）的患者接受PD-1/PD-L1单抗治疗，未区分具体突变亚型的总ORR为15.6%，DCR为50%，中位PFS为3.4个月，中位OS为18.4个月，进一步区分突变类型后发现中*MET 14*突变和*MET*基因扩增患者接受免疫治疗中位PFS没有差异（*P*=0.09）。此外既往研究提示在*MET*基因改变在肺肉瘤样癌频率更高，有研究者^[20]^报告了1例肺肉瘤样癌伴*MET* *14*突变的患者接受Nivolumab治疗，最佳疗效疾病稳定（stable disease, SD）且PFS超过6个月。

虽然上述回顾性研究报告的*MET*基因组改变接受ICIs治疗ORR不到20%，ICIs治疗有一定疗效但并不十分理想，但另一项回顾性GFPC研究^[16]^报告了较高的ORR。该研究中30例*MET* *14*突变患者接受ICIs治疗ORR能达到35.7%，DCR为71.4%，中位PFS为4.9个月，中位OS为13.4个月，研究者考虑这与纳入的PD-L1表达≥50%的患者比例较高（37%）以及ICIs治疗线数相对较少有关（63%为一线或二线治疗），这也就提示我们对于*MET*基因组改变的患者根据免疫治疗预后相关标志物筛选患者可以更精确地选择获益人群。

### 2.3 ICIs治疗*HER2*基因组改变NSCLC的疗效

*HER2*基因组改变主要为*HER2*突变及*HER2*扩增。关于*HER2*扩增的报道较少，在RTD研究中纳入的5例*HER2*扩增的患者有1例达到PR^[10]^。目前的研究主要关注于*HER2*突变患者。一项纳入122例*HER2*突变患者的回顾性研究提示77%的患者PD-L1表达小于1%（低于未选择人群），中位TMB为5.7 mut/Mb（与未选择人群无差异），26例接受免疫治疗的*HER2*突变患者ORR较低，仅为12%，DCR为42%，中位PFS为1.9个月，中位OS为10.4个月^[21]^。在IMMUNOTARGET研究中^[13]^，纳入的29例*HER2*突变患者接受ICIs单药治疗ORR为7.4%，DCR为33.3%，中位PFS为2.5个月，中位OS为20.3个月。在RTD研究中^[10]^，纳入的13例*HER2*突变的患者ICIs治疗的ORR仅为14%，中位PFS为3.4个月，中位OS为17.5个月。同样，在*MET*基因类似，在GFPC研究^[16]^中研究者报告了较高的ORR，该研究中23例*HER2*突变患者ORR为27.3%，DCR为50%，中位PFS为2.2个月，中位OS为20.4个月，研究者同样考虑较高的ORR与筛选了较高PD-L1及更早的治疗线数有关。

### 2.4 ICI治疗*RET*重排NSCLC的疗效

*RET*重排在NSCLC在总人群中突变频率为1%-2%，2019年来自美国MSKCC研究中心研究人员进行了一项针对74例*RET*重排患者的回顾性研究^[22]^提示，*RET*重排患者中81%的患者PD-L1表达低于50%，*RET*重排患者中位TMB显著低于野生型患者，仅为1.75 mut/Mb。该研究中16例*RET*重排（其中*KIF5B-RET* 10例）患者接受PD-1/PD-L1单抗和/或联合CTLA-4单抗治疗，没有患者达到完全缓解（complete response, CR）/PR，仅3例患者评价为SD，2例患者评价为non-CR/non-疾病进展（progressive disease, PD），中位PFS为3.4个月，同时并没有看到PD-L1或TMB与PFS之间的关系。同样在IMMUNOTARGET研究^[13]^中，16例*RET*重排NSCLC患者接受PD-1/PD-L1单抗治疗（其中*KIF5B-RET* 6例）仅1例患者达到PR；而在RTD研究中，4例*RET*突变患者接受免疫治疗也均对治疗无响应^[10]^。

与*MET*/*HER2*基因类似，在GFPC研究^[16]^中免疫治疗显示出来了一定疗效。研究纳入*RET*重排患者9例，2例患者PD-L1表达 > 50%，ORR为37.5%，中位PFS为7.6个月，遗憾的是研究者并没有进一步分析可能的原因，但研究者提到在这一研究中可能存在因回顾性偏倚导致的ORR被高估。

### 2.5 其他基因突变NSCLC的ICI治疗疗效

除*BRAF*、*MET*、*HER2*、*RET*等基因组改变外，还有少部分NSCLC患者具有*ROS1*、*NTRK*等驱动基因改变。在RTD研究^[10]^中纳入2例*NTRK*融合患者，1例患者达到PR；在IMMUNOTARGET研究^[13]^中共有7例*ROS1*重排患者接受PD-1/PD-L1单抗治疗，ORR为17%，研究者未报告PFS和OS等相关生存数据。目前针对这部分患者的相对研究结果较少，仍需要更多真实世界研究结果的补充。

## 3 ICIs联合其他治疗的前景与展望

结合上述研究我们可以看到，除因纳入基线PD-L1高表达患者比例高、治疗线数少的GFPC研究^[16]^外，在其他研究中PD-1/PD-L1单抗和/或联合CTLA-4单抗治疗*BRAF*基因改变的NSCLC患者ORR为20%-30%（表 1），这一水平与PD-1单抗二线治疗未选择人群的ORR类似，但对于*MET*（表 2）、*HER2*（表 3）突变患者有效率稍低，ORR分别在15%-20%、10%-15%，而伴有*RET*重排（表 4）的患者则更难从单纯免疫治疗中获益（ORR: 0%-6%）。近年来，研究者们也在不断地探索更多的治疗方案以提高治疗的有效率。

**表 1:** ICI对BRAF突变的NSCLC的疗效 Efficacy of ICIs for *BRAF*-mutated NSCLC

Study	Year	Mutation	Subtype	Treatment	ICI line	Total No.	ORR	DCR	mPFS (mon)	mOS (mon)
Dudnik, *et al*^[10]^	2018	*BRAF*	V600E	PD-(L)1 mAb	2	4	25%	-	1.5	NR
	2018	*BRAF*	nonV600E	PD-(L)1 mAb	2	5	20%	-	2.6	NR
Mazieres, *et al*^[13]^	2019	*BRAF*	NA	94% PD-1 mAb	3	43	24%	54%	3.1	13.6
Rihawi, *et al*^[14]^	2019	*BRAF*	NA	PD-1 mAb	2	11	9%	-	-	10.3
Guisier, *et al*^[16]^	2020	*BRAF*	V600E	PD-(L)1 mAb	2	26	26%	61%	5.3	22.5
	2020	*BRAF*	nonV600E	PD-(L)1 mAb	2	18	35%	53%	4.9	12.0
Dudnik, *et al*^[17]^	2018	*BRAF*	V600E	95% PD-1 mAb	2	12	25%	50%	3.7	NR
	2018	*BRAF*	nonV600E	95% PD-1 mAb	2	10	33%	56%	4.1	NR
ICIs: immune check point inhibitors; NSCLC: non-small cell lung cancer; PD-1: programmed cell death receptor-1; PD-L1: PD ligand 1; ORR: objective response rate; mPFS: median progression-free survival; mOS: median overall survival; DCR: disease control rate; NR: not reported.										

**表 2:** ICIs对*MET*突变NSCLC的疗效 Efficacy of ICIs for *MET*-mutated NSCLC

Study	Year	Mutation	Subtype	Treatment	ICI line	Total No.	ORR	DCR	mPFS (mon)	mOS (mon)
Dudnik, *et al*^[10]^	2018	*MET*	*MET* amplification	PD-(L)1 mAb	2	4	25%	-	4.9	NR
	2018	*MET*	*MET* 14 skipping	PD-(L)1 mAb	2	8	13%	-	4.0	NR
Mazieres, *et al*^[13]^	2019	*MET*	NA	94% PD-1 mAb	3	36	16%	50%	3.4	18.4
Guisier, *et al*^[16]^	2020	*MET*	*MET* 14 skipping	PD-(L)1 mAb	2	30	36%	71%	4.9	13.4
Sabari, *et al*^[19]^	2018	*MET*	*MET* 14 skipping	92% PD-(L)1 mAb	2	24	17%	46%	1.9	18.2
NA: not available.										

**表 3:** ICIs对*HER2*突变的NSCLC的疗效 Efficacy of ICIs for *HER2*-mutated NSCLC

Study	Year	Mutation	Subtype	Treatment	ICI line	Total No.	ORR	DCR	mPFS (mon)	mOS (mon)
Dudnik, *et al*^[10]^	2018	HER2	*HER2* mutation	PD-(L)1 mAb	2	7	14%	-	3.4	17.5
	2018	*HER2*	*HER2* amplification	PD-(L)1 mAb	2	5	20%	-	6.3	10.4
Mazieres, *et al*^[13]^	2019	*HER2*	NA	94% PD-1 mAb	3	29	7%	33%	2.5	20.3
Guisier, *et al*^[16]^	2020	*HER2*	*HER2* mutation	PD-(L)1 mAb	3	23	27%	50%	2.2	20.4
Lai, *et al*^[21]^	2018	*HER2*	*HER2* mutation	ICIs	-	26	12%	42%	1.9	10.4

**表 4:** ICI对*RET*突变NSCLC的疗效 Efficacy of ICIs for *RET*-mutated NSCLC

Study	Year	Mutation	Subtype	Treatment	ICI line	Total No.	ORR	DCR	mPFS (mon)	mOS (mon)
Dudnik, *et al*^[10]^	2018	*RET*	*RET* rearrangement	PD-(L)1 mAb	2	4	0%	-	3.0	14.9
Mazieres, *et al*^[13]^	2019	*RET*	*RET* rearrangement	94% PD-1 mAb	3	16	6%	25%	2.1	21.3
Guisier, *et al*^[16]^	2020	*RET*	*RET* rearrangement	PD-(L)1 mAb	2	9	38%	63%	7.6	NR
Offin, *et al*^[22]^	2019	*RET*	*RET* rearrangement	PD-(L)1 mAb	2	17	0%	38%	3.4	-

基础研究^[23]^提示BRAF TKI维莫非尼（Vemurafenib）能够通过促进主要组织相容性复合体（major histocompatibility complex, MHC）Ⅰ类分子及黑色素瘤分化抗原表达增加黑色素瘤细胞对细胞毒性T细胞的敏感性，同时阿替利珠单抗（Atezolizumab）联合靶向药物考比替尼（Cobimetinib）和维莫非尼用于治疗*BRAF* V600突变阳性的晚期黑色素瘤患者的Ⅲ期临床研究达到主要研究目的，证明了靶向BRAF TKI联合免疫治疗的临床疗效，且没有发现明显的毒性累加^[24]^。但在肺癌治疗领域，目前已有的EGFR或ALK TKI联合PD-1/PD-L1单抗治疗的临床试验中均因不良反应（如间质性肺炎、肝毒性等）发生率较大而宣告失败^[25, 26]^，尚缺乏针对少见突变靶向TKI联合ICI治疗NSCLC的证据。如果未来在相关领域进行尝试，一定要密切关注药物安全性。

此外针对突变型NSCLC，ICIs联合抗血管治疗也逐渐受到重视。基础研究提示以血管内皮生长因子（vascular endothelial growth factor, VEGF）或血管内皮生长因子受体2（VEGF receptor 2, VEGFR-2）为靶点的抗血管生成治疗可以增加T细胞向肿瘤的聚集，减少免疫微环境中免疫抑制细胞因子和调节性T细胞^[27]^。抗血管治疗与ICIs联合应用可以促进特异性T细胞迁移，促进肿瘤细胞MHC-1和PD-L1的表达^[28]^。临床方面Ⅲ期临床研究IMpower 150^[29]^已经表明免疫联合抗血管一线治疗用于经治*EGFR*突变NSCLC患者能够获得生存改善，安全性及耐受性均较好。目前尚无ICIs联合抗血管治疗治疗少见突变患者的数据，我们也期待这一治疗模式能在少见突变肺癌的领域发挥重要的作用。

## 4 局限性

首先，目前已有的临床研究多为单臂研究，无法得到OR或者HR进行荟萃分析。不同临床研究间患者基线水平不同，各项研究报告的ORR存在差异。如GFPC研究^[16]^纳入的PD-L1高表达患者多，患者治疗线数早，该研究得到各突变亚型ORR整体比其他研究高；其次，针对免疫相关标志物的研究中，大规模协作组研究结果和单中心研究结果存在差异。以*MET*基因为例，*JAMA*发表的一项研究认为突变型和野生型之间TMB无差异，而*Annals of Oncology*发表的研究认为突变型TMB较野生型低；第三，目前已有较大规模的研究以PD-1/PD-L1单抗治疗为主，随着治疗理念的更新，免疫联合抗血管等方案将发挥越来重要的作用，目前仍缺乏相关治疗模式下的在少见突变患者中的真实世界证据；第四，以上临床研究均以国外研究人群为主，我们目前仍缺乏基于我国人群的数据证据。

## 5 总结

少见突变虽然在NSCLC中发生比例较低，但患者面临预后差、靶向治疗选择少、后续治疗选择有限等挑战。通过对ICIs治疗*BRAF*、*MET*、*HER2*、*RET*等少见突变文献进行回顾，提示我们不同突变类型患者对ICIs治疗有响应，可以作为一种治疗选择的补充，但部分突变类型如*RET*重排阳性比例较低，在临床实践中应积极推动TMB、PD-L1检测以筛选最佳的获益人群，并探索更多免疫联合治疗策略进一步扩大可能的获益人群。同时也期待未来通过多中心协作组的方式进行的我国人群少见突变NSCLC患者的免疫标志物检测并开展前瞻性真实世界登记研究，为我国人群少见突变患者应用ICIs治疗提供数据支持。
